# Effects of psychosocial interventions on sexual function, quality of life, and stress in women with gynecologic cancer: a systematic review and meta-analysis

**DOI:** 10.1007/s11845-026-04439-3

**Published:** 2026-06-11

**Authors:** Esra Aydın, Handan Özcan, Sevda Karakaş, Sevda Uzun

**Affiliations:** 1 Faculty of Health Sciences, Department of Nursing, Gümüşhane University, Gümüşhane, Turkey; 2 Faculty of Health Sciences, Department of Midwifery, University of Health Sciences, Istanbul, Turkey; 3 Faculty of Health Sciences, Department of Psychiatric Nursing, Kırşehir Ahi Evran University, Kırşehir, Turkey

**Keywords:** Gynecologic cancer, Meta-analysis, Psycho-oncology, Psychosocial intervention, Quality of life, Sexual function, Stress

## Abstract

**Objectives:**

To evaluate the effects of psychosocial interventions on sexual function, quality of life, and stress levels in women with gynecologic cancer.

**Methods:**

This systematic review and meta-analysis was conducted in accordance with the PRISMA 2020 guidelines. PubMed, Web of Science, EBSCOhost, Google Scholar, and the YÖK Thesis Center were searched between June and July 2024 without year restriction. Randomized controlled and quasi-experimental studies evaluating psychosocial interventions were included. Data were synthesized using meta-analysis and narrative synthesis. Outcomes were synthesized separately for sexual function, quality of life, and stress.

**Results:**

Seven studies were included in the analysis. Psychosocial interventions significantly improved sexual function (SMD: 0.868; 95% CI: 0.021 to 1.715; Z = 2.008; p = 0.045; I^2^ = 89.97%) and quality of life (SMD: 0.709; 95% CI: 0.060 to 1.358; Z = 2.142; p = 0.032; I^2^ = 88.61%) in women with gynecologic cancer. However, psychosocial interventions did not demonstrate a significant effect on stress levels (SMD: − 1.126; 95% CI: − 2.649 to 0.397; Z = − 1.449; p = 0.147; I^2^ = 95.65%).

**Conclusion:**

This systematic review and meta-analysis indicates that psychosocial interventions are associated with improvements in sexual function and quality of life among women with gynecologic cancer, whereas no statistically significant reduction in stress was observed. Although substantial between-study heterogeneity was present, the overall evidence supports the integration of psychosocial approaches into supportive oncology nursing care.

## Introduction

Gynecologic cancers are a serious global health problem and are associated with increasing morbidity and mortality among women worldwide. Globally, gynecologic cancers are reported to be a leading cause of cancer-related death among women, following breast and lung cancer. In Turkey, gynecologic cancers are among the top 10 causes of death in women [1–3]. Treatment often constitutes a major life crisis, as it is typically prolonged and costly, may require extended hospital stays, and can be accompanied by substantial treatment-related side effects related to surgery, chemotherapy, and radiotherapy [4].

Beyond the physical burden, a diagnosis of gynecologic cancer may disrupt psychological well-being and lead to significant psychosocial difficulties. Women may experience depression, anger, fear of death, hopelessness, low self-esteem, anxiety, social isolation, communication problems, body image deterioration, reduced work and school performance, financial concerns, uncertainty about the future, and sleep and eating disorders [5, 6]. Moreover, gynecologic cancer and its treatments may adversely affect women’s sexual health and marital/partner relationships, particularly through impaired body image and a perceived decrease in sexual attractiveness [7].

Psychosocial interventions delivered during the treatment trajectory may support the biological healing process and have been associated with improvements in quality of life. Previous studies also suggest that women who receive psychosocial support may exhibit strengthened immune function and report higher life expectancy and quality of life [6, 8, 9]. Therefore, identifying psychosocial needs during diagnosis and treatment and planning timely psychosocial interventions are considered essential components of high-quality oncology care [9, 10].

Gynecologic cancer–related psychosocial interventions may reduce the psychological burden triggered by diagnosis, treatment, and body image changes, thereby strengthening daily functioning and overall well-being. A reduction in stress may improve sleep, mood, and coping resources, which can enhance quality of life; it may also decrease avoidance, anxiety, and sexual distress related to intimacy, leading to both indirect and direct benefits for sexual functioning [11, 12]. Conversely, sexual dysfunction may exacerbate psychological distress and impair quality of life, creating a self-perpetuating cycle; for example, perceived stress has been shown to mediate the association between family resilience and psychological distress [13].

Although systematic reviews and meta-analyses have examined the effects of psychosocial interventions in women with gynecologic cancer, existing syntheses often focus on a specific intervention modality (e.g., digital-only programs) or a single outcome domain (e.g., quality of life or psychological distress) [11, 14]. In contrast, the present review aims to make an original contribution to the literature by: (i) covering a broad range of psychosocial interventions without restricting delivery format (face-to-face vs online; group vs individual), (ii) addressing three key patient-reported outcomes closely linked to sexual health and psychosexual adjustment (sexual function, quality of life, and stress) through separate meta-analyses complemented by narrative synthesis, and (iii) discussing potential sources of heterogeneity in detail (e.g., intervention type, duration, measurement differences, and participant characteristics) to improve the clinical interpretability of findings.

Accordingly, this study aimed to systematically review randomized controlled and quasi-experimental studies evaluating the effectiveness of psychosocial interventions on sexual function, quality of life, and stress levels in women with gynecologic cancer and to synthesize the available evidence through meta-analysis.

## Methods

This systematic review and meta-analysis was conducted and reported in accordance with the PRISMA 2020 statement [15]. To minimize the risk of bias, the literature search, study selection, and data extraction were performed independently by four reviewers; any discrepancies were resolved through discussion and consensus. The methodological quality of the included studies was independently assessed by the reviewers.

### Inclusion and exclusion criteria

Studies were screened and selected according to the PICOS framework, as follows:

o **Population (P):** Women diagnosed with gynecologic cancer
o **Intervention (I):** Psychosocial interventions
o **Comparison (C):** Usual care or no psychosocial intervention
o **Outcomes (O):** Sexual function, quality of life, and stress (synthesized separately).
o **Study design (S):** Randomized controlled and quasi-experimental studies published in English or Turkish.

### Search strategy

The literature search was conducted between June and July 2024 using the following databases: PubMed, Web of Science, EBSCOhost, Google Scholar, and the YÖK Thesis Center. The search strategy combined relevant keywords and controlled vocabulary terms, including “gynecologic cancer,” “psychosocial intervention,” and “psychotherapeutic intervention”, using appropriate Boolean operators. Database-specific search strategies were adapted as necessary. Given the limited number of studies examining psychosocial interventions in women with gynecologic cancer, no year restriction was applied, and all available records were screened.

### Selection of studies

As a result of the database search, 3219 records were initially identified. After removal of duplicate records, 554 records remained and were screened based on titles and abstracts. Of these, 534 records were excluded for not meeting the inclusion criteria. Subsequently, 20 full-text articles were sought for retrieval; however, three reports could not be retrieved. A total of 17 full-text articles were assessed for eligibility. Of these, 10 articles were excluded due to qualitative study design (n = 5), not being published in English or Turkish (n = 4), or lacking relevant outcome data (n = 1). Finally, seven studies met the inclusion criteria and were included in the systematic review and meta-analysis.

### Data extraction of study data

A standardized data extraction form developed by the researchers was used to collect study data. Extracted information included author and publication year, study design, intervention characteristics, and main outcomes of the included studies. After full-text assessment, the eligible studies were evaluated in detail according to the inclusion and exclusion criteria. Among these, seven studies reporting the effects of psychosocial interventions on outcomes in women with gynecologic cancer were included in the systematic review and meta-analysis [6, 16–21]. The extracted data are summarized in Table 1.

**Table 1:** Characteristics and main findings of the studies included in the systematic review and meta-analysis

Author (Year)	Study design	Country	Sample size	Outcome measures	Age group	Main findings	Intervention duration	Intervention type	Quality score
Chow et al. (2020) [16]	Randomized controlled	Japan	Experimental group:102 Control group:100	SVQ (Sexual Function–Vaginal Changes Questionnaire)	Over 18 years old	Sexual function problems were less frequent in the psychoeducation group	12 weeks	Theory-oriented psychoeducation	Yes: 9/13 No: 2/13 Unclear: 1/13 Not applicable: 1/13
Aydin and Kabukcuoglu (2023) [17]	Randomized controlled	Turkey	Experimental group:34 Control group:34	SWBS (Spiritual Well-Being Scale); TSSS (Traumatic Stress Symptom Scale)	Over 18 years old	Logotherapy reduced women’s traumatic stress symptoms and enhanced posttraumatic growth and meaning in life, but had no significant effect on spiritual well-being	14 weeks	Logotherapy-based intervention	Yes: 7/13 No: 3/13 Unclear: 2/13 Not applicable: 1/13
Classen et al. (2013) [18]	Randomized controlled	Canada	Experimental group:13 Control group:14	FSDS-R (Female Sexual Distress Scale–Revised); HADS (Hospital Anxiety and Depression Scale)	Over 18 years old	Participants in online psychosocial support groups showed reduced sexual distress and stress levels	12 weeks	Online psychosocial support	Yes: 9/13 No: 2/13 Unclear: 1/13 Not applicable: 1/13
Powell et al. (2008) [19]	Randomized controlled	USA	Experimental group:43 Control group:45	QOL (Quality of Life); POMS (Profile of Mood States)	Over 18 years old	An improvement in quality of life and psychological well-being was observed in the psychosocial support group	2 weeks	Psychosocial support intervention	Yes: 9/13 No: 2/13 Unclear: 1/13 Not applicable:1/13
Li et al. (2023) [6]	Randomized controlled	China	Experimental group:49 Control group:49	FSDS-R (Female Sexual Distress Scale–Revised); QOL (Quality of Life)	Over 18 years old	Improvements in quality of life and sexual function were observed in the psychosocial intervention group	8 weeks	WeChat-based psychosocial support	Yes: 9/13 No:1/13 Unclear: 2/13 Not applicable:1/13
Kinner et al. (2018) [20]	Quasi-experimental	USA	19 women	QOL (Quality of Life); PSS (Perceived Stress Scale)	Over 18 years old	Improvements in quality of life and stress levels were observed in the psychosocial support group	10 weeks	Psychosocial support intervention	Yes:9/9
Olesen et al. (2016) [21]	Randomized controlled	Denmark	Experimental group:80 Control group:85	QOL-CS (Quality of Life–Cancer Survivors)	Over 18 years old	An improvement in quality of life was observed in the psychosocial skills program group	3 weeks	Psychosocial skills training	Yes: 9/13 No: 2/13 Unclear: 1/13 Not applicable: 1/13

### Research ethics

This study is a systematic review and meta-analysis based on previously published studies. Therefore, ethical approval and informed consent were not required.

### Assessment of methodological quality of studies

The methodological quality of the studies included in this systematic review and meta-analysis was assessed using the Joanna Briggs Institute (JBI) critical appraisal tools, which are specific to study design [22, 23]. Appropriate appraisal tools were selected according to the design of each included study. The JBI checklist consisting of 13 items was used for randomized controlled trials, and the checklist consisting of 9 items was used for quasi-experimental studies [22]. Each item was rated as “Yes,” “No,” “Unclear,” or “Not applicable,” with higher quality scores indicating a lower risk of bias and better methodological quality. Methodological quality assessment was performed independently by the reviewers, and any disagreements were resolved through discussion and consensus. In this study, the evaluation results for each study are presented in Table 1 as quality scores, where a score of 13/13 indicates high methodological quality and a low risk of bias.

### Data synthesis

Statistical analyses were performed using Comprehensive Meta-Analysis (CMA) software, version 2. Heterogeneity among studies was assessed using the Chi-squared test, Higgins I^2^ statistic, and Tau-squared statistic. An I^2^ value greater than 50% was considered to indicate substantial heterogeneity. A fixed-effects model was applied when heterogeneity was low or moderate (I^2^ ≤ 50% and p > 0.10). A random-effects model was used when substantial heterogeneity was present (I^2^ > 50% or p ≤ 0.10) [24].

In addition, the Tau-squared statistic was used to complete the assessment of variance and heterogeneity between studies. The standardized mean difference (SMD) with 95% confidence interval (CI) was used to assess the effect size associated with the same outcome with different measurement tools.

Forest plots were generated to display the pooled SMDs with corresponding 95% CIs for each outcome. Effect sizes were converted to Z-values to evaluate statistical significance. Publication bias was evaluated through visual inspection of funnel plots and the Begg and Mazumdar rank correlation test [25]. Given the small number of included studies, these assessments were of limited interpretability and should be interpreted with caution. All statistical tests were considered significant at p < 0.05.

## Results

**Fig. 1:**
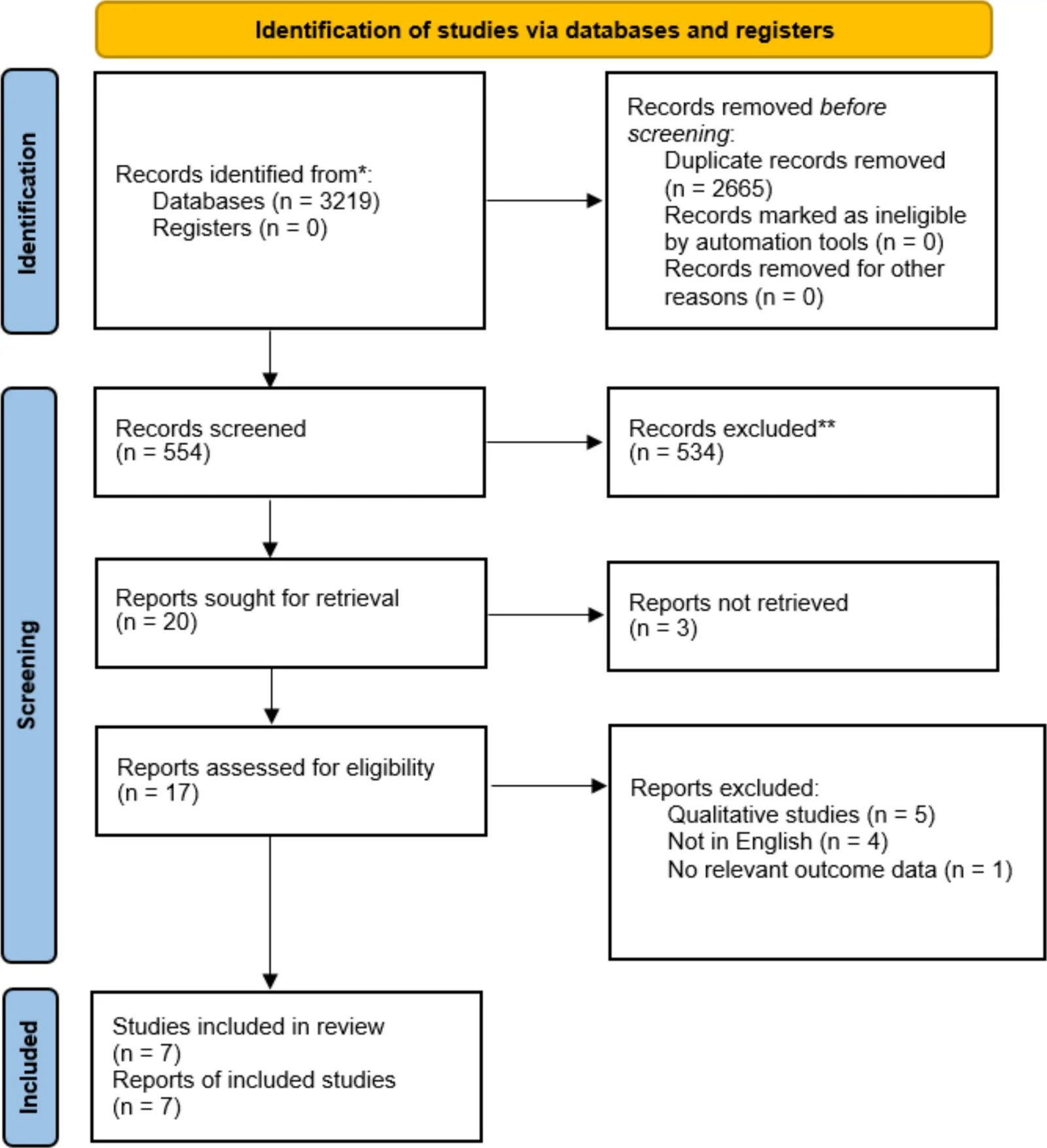
PRISMA 2020 flow diagram of study selection

The study selection process according to the PRISMA 2020 flow diagram is presented in Fig. 1.

### Results of the meta-analysis

Seven studies evaluating the effectiveness of psychosocial interventions in gynecologic cancer patients were included in the analysis [6, 16–21]. Psychosocial interventions were found to be effective in improving sexual function (SMD: 0.868; 95% CI: 0.021 to 1.715; Z = 2.008; p = 0.045; I^2^ = 89.97%) and quality of life (SMD: 0.709; 95% CI: 0.060 to 1.358; Z = 2.142; p = 0.032; I^2^ = 88.61%). However, psychosocial interventions were not effective in reducing stress levels (SMD: − 1.126; 95% CI: − 2.649 to 0.397; Z = − 1.449; p = 0.147; I^2^ = 95.65%).

Study characteristics are summarized in Table 1.

Overall, the included studies suggested that psychosocial interventions may have positive effects on patient-reported outcomes in women with gynecologic cancer, particularly sexual function and quality of life. Across studies, more than 50% of the methodological quality appraisal items were rated as “Yes,” indicating generally acceptable methodological quality.

### Meta-analysis results on the effect of psychosocial interventions on sexual function

**Fig. 2:**
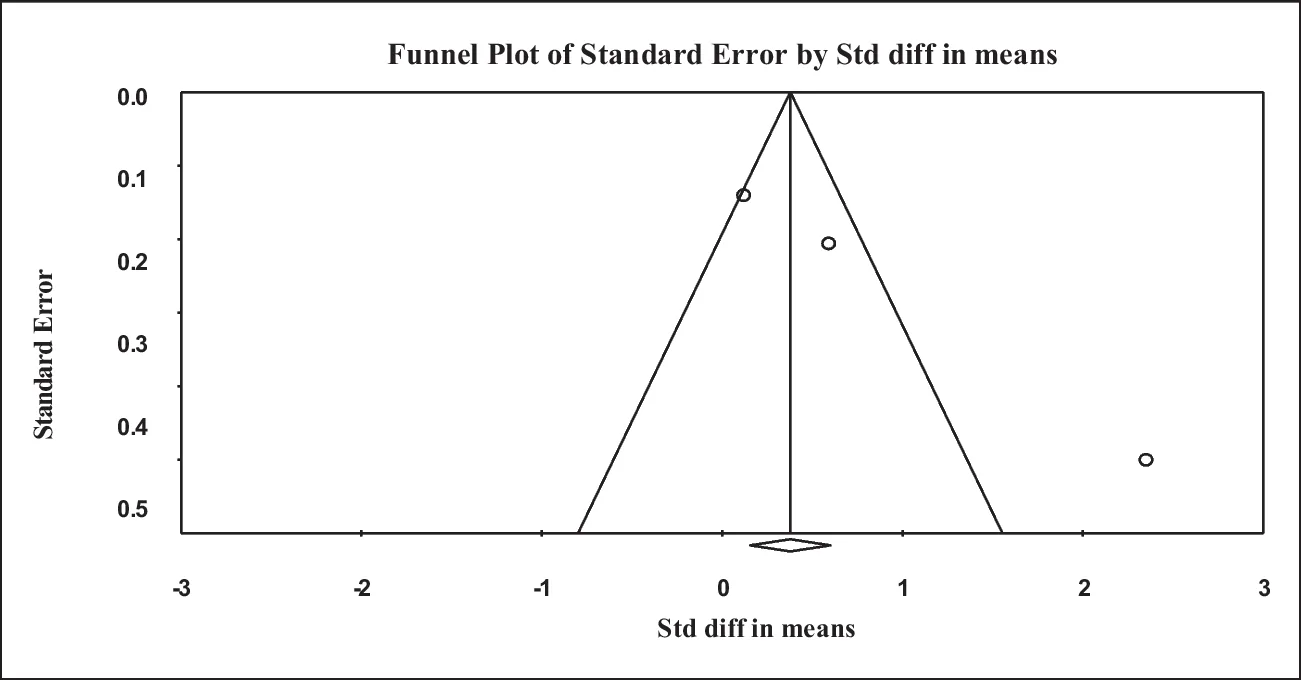
Funnel plot of studies reporting results on the effect of psychosocial interventions applied to women with gynecologic cancer on sexual function

Publication bias was assessed by visual inspection of the funnel plot (Fig. 2) and the Begg and Mazumdar rank correlation test. Given the small number of included studies (n = 3), these assessments were of limited interpretability and should be interpreted with caution.

**Fig. 3:**
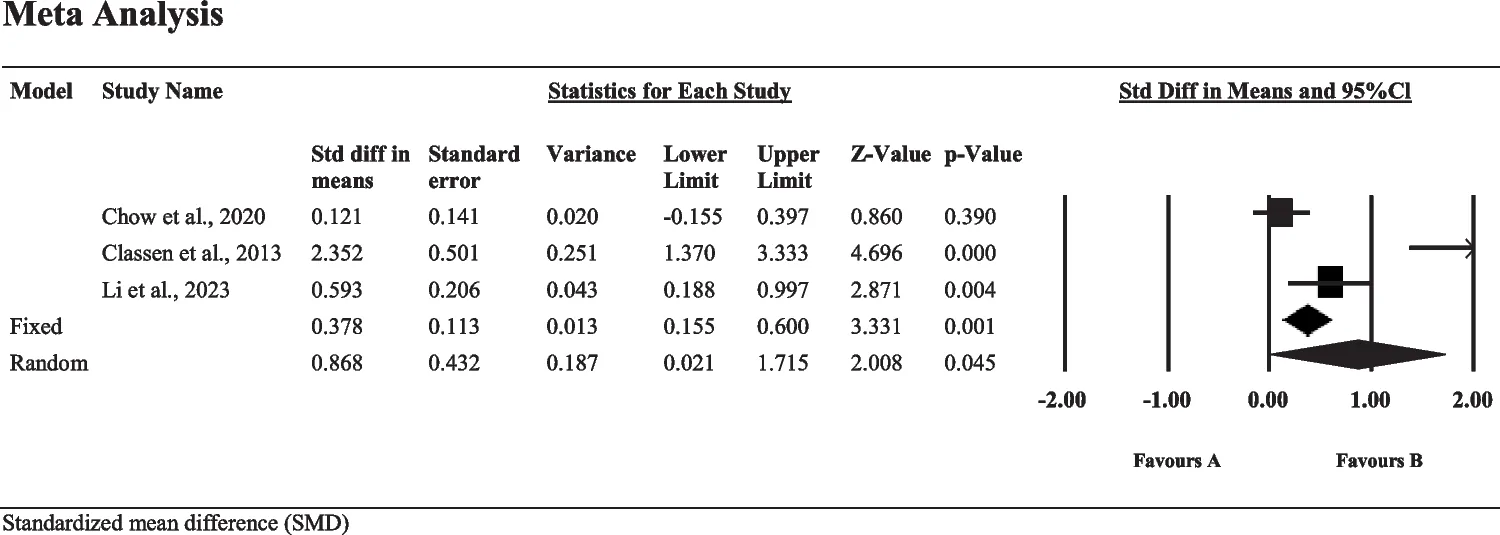
Forest plot for the effect of psychosocial interventions on sexual function between intervention and control groups

Figure 3 shows the effect sizes, standard error, variance, lower and upper limits, and forest plot of 3 studies on the effect of psychosocial interventions applied to women with gynecologic cancer on sexual function. In the meta-analysis based on the findings of these studies, it was found that psychosocial interventions applied to women with gynecologic cancer were effective on sexual function (SMD: 0.868; 95% CI: 0.021 to 1.715; Z = 2.008; p = 0.045). The collected data showed that psychosocial interventions applied to women with gynecologic cancer had an overall significant effect on sexual function in favor of the intervention group, and a high level of heterogeneity was found between studies (I^2^ = 89.97%).

### Meta-analysis results on the effect of psychosocial interventions on quality of life

**Fig. 4:**
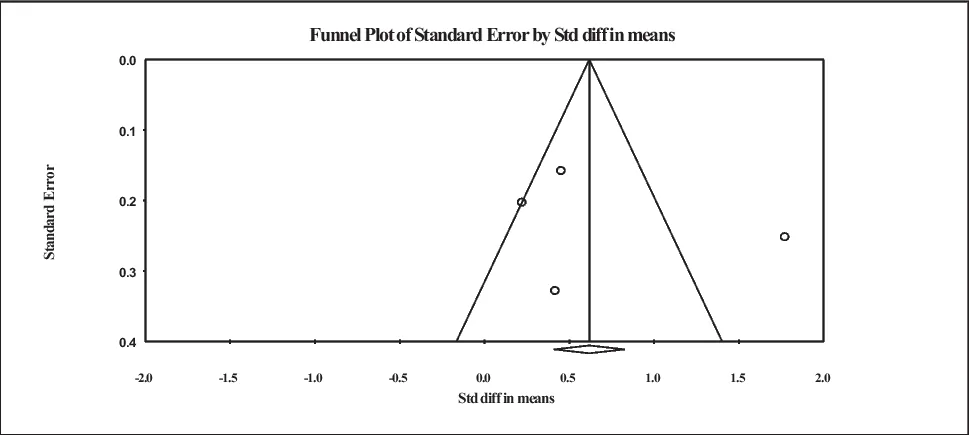
Funnel plot of studies reporting results on the effect of psychosocial interventions applied to women with gynecologic cancer on quality of life

Publication bias was assessed by visual inspection of the funnel plot (Fig. 4) and the Begg and Mazumdar rank correlation test. Given the small number of included studies (n = 4), these assessments were of limited interpretability and should be interpreted with caution.

**Fig. 5:**
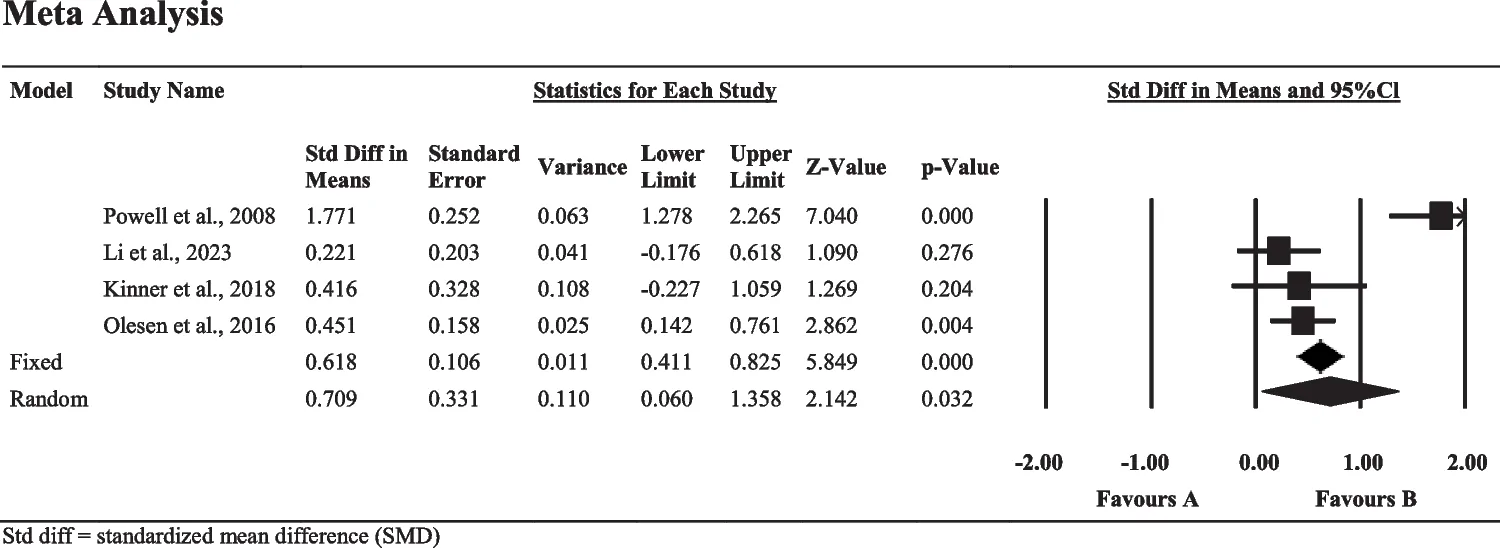
Forest plot for the effect of psychosocial interventions on quality of life between intervention and control groups

Figure 5 shows the effect sizes, standard error, variance, lower and upper limits, and forest plot of 4 studies on the effect of psychosocial interventions applied to women with gynecologic cancer on quality of life. In the meta-analysis based on the findings of these studies, it was found that psychosocial interventions applied to women with gynecologic cancer were effective on quality of life (SMD: 0.709; 95% CI: 0.060 to 1.358; Z = 2.142; p = 0.032). The collected data showed that psychosocial interventions applied to women with gynecologic cancer had an overall significant effect on quality of life in favor of the intervention group and a high level of heterogeneity was detected between studies (I^2^ = 88.61%).

### Meta-analysis results on the effect of psychosocial interventions on stress levels

**Fig. 6:**
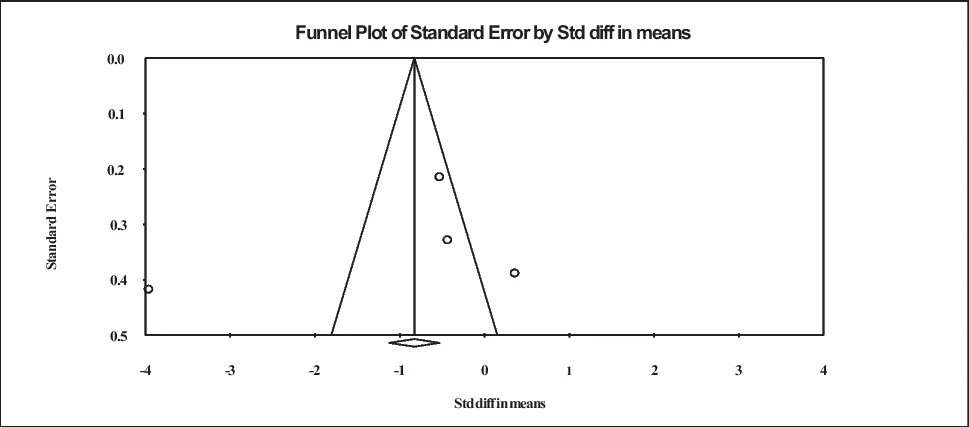
Funnel plot of studies reporting results on the effect of psychosocial interventions applied to women with gynecologic cancer on stress levels

Publication bias was assessed by visual inspection of the funnel plot (Fig. 6) and the Begg and Mazumdar rank correlation test. Given the small number of included studies (n = 4), these assessments were of limited interpretability and should be interpreted with caution.

**Fig. 7:**
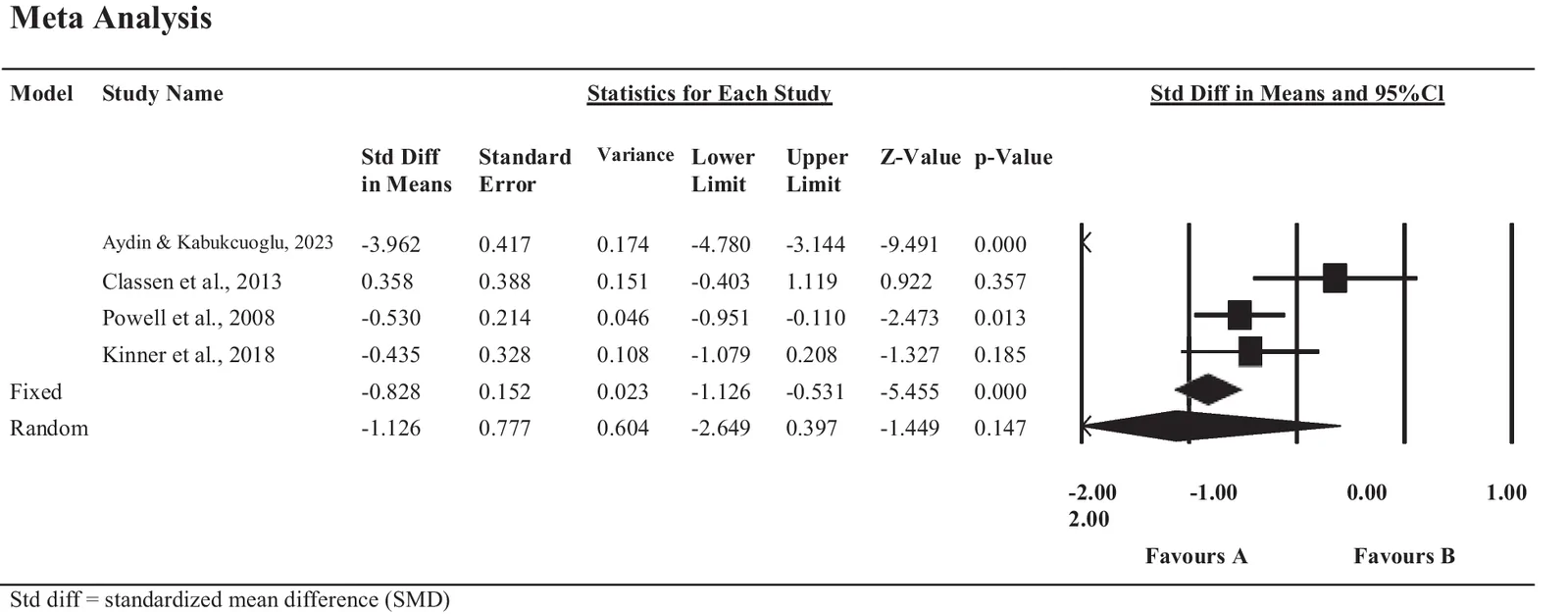
Forest plot for the effect of psychosocial interventions on stress levels between intervention and control groups

Figure 7 shows the effect sizes, standard error, variance, lower and upper limits, and forest plot of 4 studies on the effect of psychosocial interventions on stress in women with gynecologic cancer. In the meta-analysis based on the findings of these studies, it was found that psychosocial interventions applied to women with gynecologic cancer were not effective on stress levels (SMD: −1.126; 95% CI: −2.649 to 0.397; Z = −1.449; p = 0.147). The collected data showed that psychosocial interventions applied to women with gynecologic cancer did not have an overall significant effect on stress levels in favor of the intervention group, and a high level of heterogeneity was detected between studies (I^2^ = 95.65%).

## Discussion

This systematic review and meta-analysis synthesized evidence from randomized controlled and quasi-experimental studies evaluating the effects of psychosocial interventions on sexual function, quality of life, and stress in women with gynecologic cancer. Overall, psychosocial interventions were associated with significant improvements in sexual function and quality of life, whereas no statistically significant pooled effect was observed for stress. Importantly, rather than combining these outcomes into a single composite endpoint, we conducted separate outcome-specific meta-analyses and interpreted pooled estimates alongside study-level patterns to enhance clinical interpretability.

This review extends prior syntheses by covering a broad range of psychosocial interventions without restricting delivery mode (face-to-face vs online; group vs individual) and by jointly addressing three closely related patient-reported outcome domains relevant to psychosexual adjustment. Nevertheless, substantial between-study heterogeneity was observed across outcomes, likely reflecting differences in intervention content and intensity, measurement tools, and follow-up timing. Therefore, findings—particularly for stress—should be interpreted cautiously, and future studies using more standardized intervention protocols and outcome measures are needed to clarify which components are most effective for specific patient needs.

The high between-study heterogeneity observed across outcomes (I^2^ > 88%) may be driven by differences in measurement tools (e.g., SVQ vs. FSDS-R for sexual function; PSS vs. HADS/TSSS for stress), intervention content and intensity, delivery formats (face-to-face vs. online), participant characteristics (e.g., cancer stage and age), and follow-up duration. These factors underscore the need for more standardized protocols in future research to reduce variability and enhance comparability. Taken together, these sources of heterogeneity may help explain the variability in effect estimates, particularly for stress. Because of the small number of included studies for each outcome, publication bias assessments were of limited interpretability and should be interpreted with caution. In addition, the limited number of studies restricted further analyses to explore potential sources of heterogeneity.

Overall, these findings support integrating structured psychosocial care into supportive oncology nursing, with particular attention to sexual health and quality-of-life outcomes.

Gynecologic cancer treatments—including surgery, chemotherapy, and radiotherapy—are frequently accompanied by distressing symptoms and a prolonged treatment trajectory, which may contribute to impaired quality of life. In the present meta-analysis, psychosocial interventions demonstrated a statistically significant beneficial effect on quality of life. Consistent with our findings, Yen et al. reported positive effects of psychosocial interventions on quality of life in women undergoing gynecologic cancer treatment [14]. In contrast, Graetz et al. found no significant improvement in quality of life following a 30-day web-based psychosocial intervention [26]. Such discrepancies may reflect differences in intervention content, duration, delivery modality, and eligibility criteria. Accordingly, further well-designed studies using standardized intervention protocols and validated outcome measures are warranted to clarify which components are most effective for improving quality of life.

Gynecologic cancers and their treatments may negatively affect body image and reproductive function, thereby contributing to sexual dysfunction. The pooled analysis indicated that psychosocial interventions were associated with improvements in sexual function. Shi et al. reported improvements in sexual satisfaction and sexual quality of life following a nurse-led psychosocial intervention [27]. In addition, Ask et al. concluded in their meta-analysis that psychosocial interventions positively influenced sexual health outcomes among patients with pelvic cancers [11]. Classen et al. also reported that a 12-week psychosocial intervention reduced sexual distress and improved sexual functioning in women receiving treatment for gynecologic cancer [18]. Collectively, these findings suggest that psychosocial interventions may play an important role in supporting sexual health and mitigating sexual problems in this population.

In contrast, this meta-analysis did not identify a statistically significant pooled effect of psychosocial interventions on stress levels. Siwik et al. reported that psychosocial support strengthened coping abilities and reduced stress and depression among women with gynecologic cancer [28]. However, Petzel et al. found that a 60-day psychosocial support intervention did not yield a significant improvement in patient-reported mental health outcomes [29]. Similarly, Kinner et al. reported limited effects of a 10-week web-based psychosocial support intervention on emotional well-being [20]. These inconsistencies across studies may be attributable to differences in stress measurement tools, baseline psychological status, intervention content and intensity, and follow-up duration. In addition, some studies assessed related but non-identical constructs (e.g., perceived stress vs traumatic stress symptoms), which may have contributed to the variability in effect estimates. Moreover, the very high heterogeneity observed in the stress analyses necessitates cautious interpretation. Therefore, high-quality studies with standardized protocols, adequate sample sizes, and longer follow-up are needed to more clearly determine the effects of psychosocial interventions on stress in women with gynecologic cancer.

### Implications for nursing practice

This systematic review and meta-analysis indicates that psychosocial interventions can provide meaningful benefits for women with gynecologic cancer, particularly by improving sexual function and quality of life. Given that sexual health–related concerns are often insufficiently recognized in oncology care and may not be systematically incorporated into routine assessments, these findings suggest that nursing care should not be limited to physical symptom management but should be supported by planned psychosocial components throughout the treatment trajectory.

In clinical practice, oncology nurses should regularly assess sexual distress, body image, and relationship/partner problems using brief and valid measurement tools, and address sexuality and intimacy through culturally sensitive communication. Based on identified needs, nurses may deliver structured psychosocial interventions—such as theory-informed psychoeducation, skills-based counseling, and technology-supported programs—or coordinate referral to appropriate services.

Although no statistically significant pooled effect was observed for stress and between-study heterogeneity was substantial, individualized care planning remains essential. Tailoring the content and intensity of interventions according to baseline psychological status, treatment phase, and patient-reported priorities may enhance clinical benefit and help identify those most likely to benefit. Overall, integrating evidence-informed psychosocial care into oncology nursing practice may strengthen patient-centered care by supporting sexual health and improving quality of life in women living with gynecologic cancer.

### Limitations

This systematic review and meta-analysis has several limitations that should be considered when interpreting the findings. First, some included studies had relatively small sample sizes and lacked blinding, which may have reduced methodological rigor and the overall strength of the evidence. Second, substantial heterogeneity was observed across the meta-analyses, likely due to variations in study design, types and duration of psychosocial interventions, outcome measurement tools, and patient characteristics. Third, the limited number of studies per outcome restricted further analyses to explore potential sources of heterogeneity. Finally, because of the small number of included studies, publication bias assessments were of limited interpretability. Therefore, the findings should be interpreted with caution.

## Conclusion

This systematic review and meta-analysis demonstrated that psychosocial interventions were associated with significant improvements in sexual function and quality of life in women with gynecologic cancer, whereas no statistically significant pooled effect was observed for stress. These findings highlight the potential role of psychosocial interventions as an important component of comprehensive cancer care. Nevertheless, the limited number of high-quality studies and substantial heterogeneity across outcomes indicate a need for further well-designed randomized controlled and quasi-experimental studies to strengthen the evidence base. Nurses play a key role in identifying psychosocial needs arising from gynecologic cancer and should integrate appropriate psychosocial interventions into patient-centered care to support sexual health and overall quality of life.

## Data Availability

The data that support the findings of this study are available from the corresponding author upon reasonable request. As this study is a systematic review and meta-analysis based on previously published studies, all analyzed data are available in the published articles cited in the manuscript.
